# Raising awareness of riverine populations in the Brazilian Amazon about MeHg intoxication in *APOE*4 carriers: cardiovascular risk and potential benefit of native selenium diets

**DOI:** 10.3389/ftox.2025.1571658

**Published:** 2025-05-08

**Authors:** Camila G. M. Carvalho, Patrícia Marçal Da Costa, Luana M. Rocha Souza, Vitória K. Félix Monteiro, Jacqueline I. Alvarez-leite, Maria Elena Crespo-Lopez, Reinaldo B. Oriá

**Affiliations:** ^1^ Laboratory of the Biology of Tissue Healing, Ontogeny and Nutrition Biomedicine Center, School of Medicine, Federal University of Ceará, Fortaleza, Brazil; ^2^ Laboratory of Atherosclerosis and Nutritional Biochemistry, Department of Biochemistry and Immunology, Federal University of Minas Gerais, Belo Horizonte, Brazil; ^3^ Laboratory of Molecular Pharmacology, Institute of Biological Sciences, Federal University of Para, Belem, Brazil

**Keywords:** methylmercury, apolipoprotein E4, metabolic syndrome, obesity, selenium, antioxidants, DIETS

## Introduction

The rapid expansion of illegal gold mining in the Amazon has caused severe social and environmental issues, particularly mercury (Hg) contamination, threatening biodiversity and the health of local communities (Domingues et al., 2024). Amazonian riverside populations have historically been exposed to chronic methylmercury (MeHg) levels due to ingestion of contaminated fish from water reservoirs (Crespo-Lopez et al., 2021).

Anthropogenic activities in the Amazon have driven MeHg, a highly toxic organic form of Hg, to bioaccumulate in the trophic chain, mainly in fish used as subsistence food in riverside communities (Nyholt et al., 2022), raising public health concerns, especially for vulnerable populations. The Amazonian riverside populations are isolated communities far from urban areas, with poor health service access. They strongly rely on the river to sustain fishing and obtain dietary proteins.

The awareness that MeHg has multisystemic effects, apart from its well-known neurological toxicity, is of utmost importance since accumulating evidence points to chronic MeHg intoxication as a culprit of increasing cardiovascular risk in preclinical and clinical studies (Ginsberg et al., 2014; Lopes-Araújo et al., 2023). MeHg exposure significantly increases the risk of fatal and non-fatal cardiovascular complications, with tipping points as low as 1 μg/g hair Hg (Hu et al., 2021a). MeHg deleterious effects on cardiovascular and atherosclerotic risk may be aggravated by the obesity epidemics occurring even in the Amazon region (Silva et al., 2021). A recent increase in non-communicable diseases (NCDs) like hypertension, diabetes, and obesity among Amazonian riverine populations, comparable to urban Brazil, has drawn academic attention. Arrifano et al. (2018) linked the APOE4 allele to hypertension and altered fasting blood glucose in these communities (Arrifano et al., 2018a).

Human apolipoprotein E (ApoE) is a 299-amino acid long protein that affects cholesterol reverse transport and metabolism. The combination of two mutations at the ApoE gene (*APOE*) originates the three main alleles *APOE2, APOE3*, and *APOE4* (Abondio et al., 2023). The *APOE4* allele is a well-known risk factor for cardiovascular diseases worldwide due to its effect on rising cholesterol levels and pro-inflammatory mediators (McMaster et al., 2024).

Selenium (Se) supplementation has been found to reduce plasma total cholesterol and LDL levels and ameliorate HDL levels in ApoE-deficient mice (Guo et al., 2020). Se is a micronutrient with potent antioxidant properties and a well-recognized cardioprotective element. It may favor long-term cardiovascular protection if incorporated adequately into a daily diet.

In this opinion paper, we summarized the up-to-date scientific literature on the effects of MeHg intoxication and cardiovascular risk when compounded with *APOE4*, in the Brazilian Amazon region. Our group recently found that *APOE4* may influence Hg intoxication levels (Arrifano et al., 2018b). We also highlight the importance of native Se-enriched diets to benefit cardiovascular health in people with the *APOE4* genetic trait living under MeHg endemic intoxication.

## MeHg and APOE4 potential interaction and cardiovascular effects

MeHg is a neurotoxic pollutant with a broad range of adverse health effects. Beyond its well-known neurotoxicity, other lines of research have highlighted its detrimental impact on the cardiovascular system (Moreira et al., 2012). Exposure to MeHg can cause cardiac remodeling, leading to increased muscle mass, altered rhythm, and reduced contractile function. These changes are associated with mitochondrial dysfunction, as the exacerbated production of reactive oxygen species (ROS) causes cellular damage and impairs cardiac function (Santos Ruybal et al., 2020).

Chronic MeHg intoxication in young *APOE* knockout (ko) mice may aggravate dyslipidemia and lead to higher lipid peroxidation levels. Furthermore, ApoE deficiency, independently of MeHg intoxication, elevated systemic lipid parameters (Roque et al., 2021). MeHg intoxication worsens cardiovascular risk, aggravating atherosclerosis in wild-type and *APOE* ko mice (Silva et al., 2021). Interestingly, MeHg can affect phospholipase-D (PLD) in vascular endothelial cells through constitutive phospholipase-A2 (PLA2) pathway and the cyclooxygenase and lipoxygenase-driven eicosanoids by oxidative stress (Sherwani et al., 2013).

In a recent review, it has been discussed how different isoforms of ApoE, in particular the ApoE4, can affect the progression of atherosclerosis in patients with periodontal disease (Pereira et al., 2019). According to Arrifano and collaborators, 65% of *APOE4* carriers had altered fasting blood sugar levels and/or systemic arterial hypertension (Ginsberg et al., 2014). More studies are needed to show the interactions between circulating lipids, diet, and MeHg intoxication and the interactions that play critical roles in the risk of chronic diseases later in life (Roque et al., 2021).

### Se-rich foods as adjuvants to reduce MeHg toxicity

Se is an essential micronutrient with antioxidant properties. It can protect against MeHg toxicity by forming stable complexes with the metal, reducing its bioavailability, and promoting excretion (Ferreira et al., 2021; Liu et al., 2019). Furthermore, Se contributes to the mitigation of inflammation and oxidative stress, crucial elements in the progression of cardiovascular diseases (Zhang et al., 2023). Adequate physiological Se plasma levels vary from 90 to 120 μg/L (Radomska et al., 2021). Such range values may change depending on the need for biological protection against Hg ingestion.

The Recommended Dietary Allowance (RDA) value for children aged 1–3 years is 20 µg/day, and from 4 to 8 years is 30 µg/day for both sexes. Men and women aged between 14 and 70 need 55 µg/day, pregnant women 60 µg/day, and lactating mothers 70 µg/day, presenting the highest intake needs. The tolerable upper intake level (UL) and the maximum daily intake for all adults over 19 years of age and pregnant and lactating women are 400 µg/day Se, considering selenosis as the adverse effect (Barchielli et al., 2022; Zhang et al., 2019). Se intake comes from food, the content of which depends on its accumulation in the soil and plants. In general, dietary Se intake in Brazil varies from slightly low to adequate or above RDA (between 54.4 and 142 μg/day), depending on the studied region (Fávaro et al., 1997).

Experimental results in rats showed that the Se-deficient diet decreased serum GSH-PX activity, which caused severe cardiac dysfunction in the animals. Suggesting a fine relationship between a Se-based diet and protection against cellular oxidative stress (Zhang et al., 2019). Low Se levels were significantly associated with decreased performance in neurological tests (Shahar et al., 2010). A diet incorporating one Brazil nut daily, providing approximately 288 µg of Se, for 6 months has been shown to enhance cognitive performance in patients by restoring optimal selenium levels in the body (Rita Cardoso et al., 2016).

Proteins containing at least one selenocysteine (SeC) residue are termed selenoproteins and play crucial physiological roles, primarily centered on maintaining cellular redox balance. Se deficiency results in reduced expression of potassium channels, STAT3 activity, and mitochondrial function (Leszto et al., 2024). Activation of STAT3 has been identified as a key cardioprotective signal in animal studies and humans (Kleinbongard, 2023).

MeHg induces oxidative stress and inflammation, leading to endothelial dysfunction and decreased antioxidant defenses. MeHg also has a high affinity for Se-based compounds, leading to decreased antioxidant activity of Se-containing enzymes, such as glutathione peroxidase (Takahashi and Shimohata, 2019). Several lines of evidence point out oxidative stress as a key driving molecular mechanism in MeHg-induced intoxication (Antunes dos Santos et al., 2018). Such effects may be associated with increased Rho-kinase activity (a class of GTPases) that negatively modulates the endothelial nitric oxide synthase (eNOS) function, reducing nitric oxide bioavailability in the vasculature, contributing to vasoconstriction and increased systemic blood pressure (Islam et al., 2016).

There has been growing interest in how Se may help reduce the harmful effects of mercury exposure from dietary sources in humans (Tinggi and Perkins, 2022). Dietary Se intake may mitigate Hg toxicity, with implications for human health, particularly for high-risk groups in a population. The protective Se effect against MeHg toxicity is considered a hot topic (Wang et al., 2017). Selenoenzymes generally prevent and reverse oxidative damage in the brain and neuroendocrine tissues. In contrast, inhibition of selenoenzyme activity in these tissues appears to cause the toxic and pathological effects of MeHg (Ralston and Raymond, 2010). The covalent bond between Hg and Se is markedly strong, making MeHg an effective Se scavenger. This interaction significantly involves the function of selenoenzymes, essential for several biochemical interactions (Fávaro et al., 1997). The selenocystine, for example, is a component found in some selenoenzymes, which are the functional proteins that utilize this amino acid. The effect of selenocystine (SeCys2) against MeHg cytotoxicity in HepG2 cells acts reducing the cytotoxicity of MeHg (Wang et al., 2017).

A dysfunction resulting from MeHg competition for the active sites of selenoenzymes, especially in fetal neuroendocrine tissues with low Se reserves, can lead to adverse effects and, in severe cases, death (Branco et al., 2022). Se supplementation can act as a chelator, accelerating MeHg clearance and restoring the activity of selenoenzymes, thus preventing neurotoxic damage.

### Native Se-based foods as nutritional interventions against MeHg poisoning

The Se content was estimated in several foods consumed in Brazil from different regions; as a result, it was discovered that foods considered traditional in the Brazilian diet, such as rice, beans, wheat flour, corn, and cassava flour, had low mineral levels. Food habits in the Manaus region differ significantly from those of other regions of the Amazon or even outside the Amazon, such as Mato Grosso and Santa Catarina. Both states consume rice and beans, but the protein ratio may vary depending on the local culture (Ferreira et al., 2002).

The Tapajós river region in the Brazilian Amazon presents a wide variation in Se levels in the local population, leading to blood levels ranging from 142 to 2,447 μg/L (Lemire et al., 2009). The average normal blood level of Se in many studies was 139 μg/L (Hadrup and Ravn-Haren, 2021). Eating Se-rich foods, such as Brazil nuts, may significantly contribute to mitigating the adverse effects of MeHg, especially in Amazonian populations exposed to high levels of this heavy metal (Takahashi and Shimohata, 2019).

Se levels in the Amazonian riverside population may vary considerably and are influenced by household location and time of year factors. Brazil nuts from Amazonas and Amapá have higher Se contents than those from other Amazon states (Silva Junior et al., 2017).

One of the primary sources of Se comes from the Brazil nut (*Bertholletia excelsa*), is a native Se-rich food (Macan et al., 2022), widely distributed in the Brazilian Amazon with Se levels up to 512 mg/kg, with higher levels obtained from trees with lower fruit production (Gomes et al., 2024). Cassava, rice, beans, and some local fruits may also be Se food sources, relying on Se-rich soils. In addition, meat, chicken, eggs, and vegetables can significantly contribute to the daily intake of this micronutrient to Amazonian populations, depending on local availability (Monteiro and Verly Júnior, 2023).

Incorporating Se into proteins by replacing sulfur in amino acids like methionine is a relevant biochemical mechanism for its bioaccumulation in animal and plant tissues. Protein-rich foods have high Se levels, but their concentration varies by animal species, diet, and region of origin (Minich, 2022).

Although animal-based foods are important Se sources, some plants, such as cruciferous vegetables (broccoli) and garlic (*Allium sativum*), may show low to moderate levels and become relevant dietary sources. Brewer’s yeast is also recognized for its Se content. In regions with Se-rich soil, cereals such as wheat can have considerable Se levels, contributing to the mineral intake through bread and other derived products (Hu et al., 2021b). Given the above, a Se-rich diet based on Amazonian traditional eating habits may help protect against cardiovascular diseases in MeHg-exposed populations, especially for *APOE4* carriers.

Factors like climate change, agricultural practices, and meal preparation methods also can influence Se levels in food (Lu et al., 2024). Although Se is essential for humans, high oral exposure can cause acute toxicity. Toxic Se blood levels may be fatal when levels exceed 300 μg Se/L (normal level: 100 μg/L), especially with prolonged exposure. Most fatal cases of Se intoxication are related to the ingestion of gun-bluing agents containing selenous acid (Hadrup and Ravn-Haren, 2020). Caution is needed to avoid long-term high Se supplementation that may cause undesirable chronic toxic effects (selenosis), such as dermatological, gastrointestinal, neurological symptoms, and multiorgan damage. We do not know whether ApoE isoforms could influence the absorption of Se from diets. Notably, *APOE4* was associated with distinct clinical outcomes following micronutrient supplementation in Brazilian shantytown children (Mitter et al., 2012).

*APOE* can regulate selenoprotein P levels, a key Se transport protein, by interacting with its heparin-binding sites (Kim et al., 2025). Interestingly, in a rural study enrolling elderly Chinese, *APOE4* carriers showed lower nail Se levels than non-carriers, even after controlling for estimated dietary Se consumption (Gao et al., 2009), supporting that *APOE* alleles may have specific effects in Se metabolism.

In conclusion, the interplay between MeHg and Se in *APOE4* carriers may be critical in determining long-term cardiovascular outcomes, which may have public health consequences. While MeHg and *APOE4* pose significant risks to cardiovascular health, Se can act as a protective agent by mitigating oxidative stress and inflammation. Dietary interventions, particularly those focused on increasing the consumption of Se-rich foods, can be a valuable strategy to reduce the adverse effects of MeHg exposure in genetically-risk individuals. Although Se might help reduce MeHg intoxication in *APOE4* carriers, caution should be taken to avoid Se toxicity. In addition, more studies are needed to define adequate Se safety levels following MeHg intoxication.
